# The effect of different suturing materials for abdominal fascia wound closure on the collagen I/III expression ratio in rats

**DOI:** 10.1016/j.amsu.2020.10.033

**Published:** 2020-10-23

**Authors:** Imam Sofii, Wisnu Dipoyono, Heryu Prima, Yessy Martha Sari, Aditya Rifqi Fauzi, Gunadi

**Affiliations:** a Digestive Surgery Division, Department of Surgery, Faculty of Medicine, Public Health and Nursing, Universitas Gadjah Mada/Dr. Sardjito Hospital, Yogyakarta, 55281, Indonesia; b Pediatric Surgery Division, Department of Surgery, Faculty of Medicine, Public Health and Nursing, Universitas Gadjah Mada/Dr. Sardjito Hospital, Yogyakarta, 55281, Indonesia

**Keywords:** Abdominal fascia, Monofilament non-absorbable, Multifilament absorbable, Incisional hernia, *Collagen I/III* ratio, *Rattus norvegicus*

## Abstract

**Background:**

Incisional hernia is a frequent complication of abdominal wall incision and has a high rate of recurrence. Most of the studies stated that non-absorbable sutures decreased incisional hernia incidences, but some stated otherwise. We aimed to compare the *collagen type I/III* ratio between monofilament non-absorbable sutures and multifilament absorbable sutures for abdominal fascia closure in Wistar albino rats.

**Methods:**

Forty rats were divided into four groups. Groups 1 and 3 were sutured with monofilament non-absorbable (*polyvinylidene fluoride*). Groups 2 and 4 were sutured with multifilament absorbable (*polyglycolide*). Then, groups 1 and 2 were euthanized on day 4 (POD 4), while groups 3 and 4 were euthanized on day 7 (POD 7). Samples of fascia (1 × 0.5 cm) were taken for analysis. *Collagen I/III* ratios were measured using immunohistochemistry staining methods.

**Results:**

While the expression of *collagen I* was not significantly different between monofilament non-absorbable and multifilament absorbable at POD 4 and 7 (*p* = 0.45 and 0.81, respectively), the expression of *collagen III* reached a significant level with *p*-values of 0.0003 and 0.0004 for POD 4 and 7, respectively. Moreover, the *collagen I/III* ratio was also significantly different between the two groups either at POD 4 (0.88 ± 0.23 *vs.* 0.53 ± 0.08; *p* = 0.0003) and 7 (1.77 ± 0.65 *vs.* 1.03 ± 0.28; *p* = 0.004).

**Conclusions:**

Monofilament non-absorbable sutures show a significantly higher *collagen I/III* ratio than multifilament absorbable sutures for abdominal fascia closure in rats. Our findings imply that the usage of monofilament non-absorbable sutures might have a beneficial effect on decreasing the incisional hernia occurrence.

## Highlights

• One of the most important factors that influences the occurrence of incisional hernia is suturing material.
• Collagen is a major component of the extracellular matrix and plays an important role in maintaining tissue elasticity and tensility.
• The usage of monofilament non-absorbable sutures might have a beneficial effect on decreasing the incisional hernia occurrence.

## Abbreviations

CI: Confidence interval
POD: Postoperative day

## 1 Background

Incisional hernia is a frequent complication of post-abdominal wall incisions. In various meta-analysis studies, the incidence rate of incisional hernia at 23.8 months post-abdominal incision was 12.8%, with an incidence of 69% reported in high-risk patients in long-term follow-up. The occurrence of an incisional hernia has an important impact on patients’ quality of life. The incidence of postoperative recurrence of incisional hernia reached above 30% [1].

The costs incurred for handling incisional hernias are notably high, such as in France, where they reached € 7089 in 2011. Therefore, reducing the incidence rate of incisional hernia requires optimal closure of the post-incision abdominal wall to save the costs and use of the health facilities and reduce post-operative disability [1]. In America, the incidence of cumulative incisional abdominal hernias reaches 2–20%, with an estimated annual cost of billions of US dollars [2]. In addition to an incisional hernia, wound dehiscence also contributes to postoperative morbidity and mortality [3].

One of the most important factors that influences the occurrence of incisional hernia is suturing material. A meta-analysis stated that absorbable sutures increase the occurrence of incisional hernia [4]. In 1993, a study also confirmed that incisional hernia occurred more often in patients sutured by monofilament material [5].

Currently, there is strong evidence of connective tissue changes, such as wound healing disorders, that have an impact on the occurrence of an incisional hernia. This study focuses more on the biochemical ratio of collagen types I and III. Collagen is a major component of the extracellular matrix and plays an important role in maintaining tissue elasticity and tensility. Collagen type I is a strong collagen that is widely distributed in the human body including the fascia, skin, ligaments, and fibrous tissue and is responsible for the mechanical resistance of the tissues, while collagen type III appears at the beginning of wound healing with fewer amounts and is less strong. During the healing process, collagen type III in the wound is replaced by the type I collagen, which is stronger; therefore, the collagen ratios of types I and III play an important role as parameters in wound healing postoperatively [6]. We aimed to compare the *collagen type I/III* ratio between monofilament non-absorbable sutures and multifilament absorbable sutures for abdominal fascia closure in Wistar albino rats.

## 2 Material and methods

### 2.1 Animal models

Forty male *Rattus novergicus*, aged 2–3 months, weighing 170–200 g were isolated under standard conditions for 7 days. Rats that were sick, had an infection during the procedures, or died during the procedures were excluded from this study. The work has been reported in line with the ARRIVE statement [7].

### 2.2 Experimental procedures

**Fig. 1:**
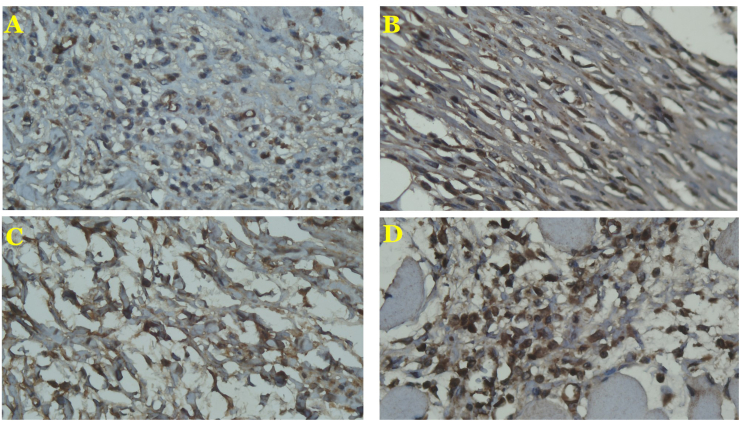
The expressions of *collagen I* on POD 4 in the monofilament non-absorbable (A) and multifilament absorbable group (B) and POD 7 in the monofilament non-absorbable (C) and multifilament absorbable group (D). The expressions of *collagen I* were similar between groups on POD 4 (*p* = 0.45) and 7 (*p* = 0.81).

**Fig. 2:**
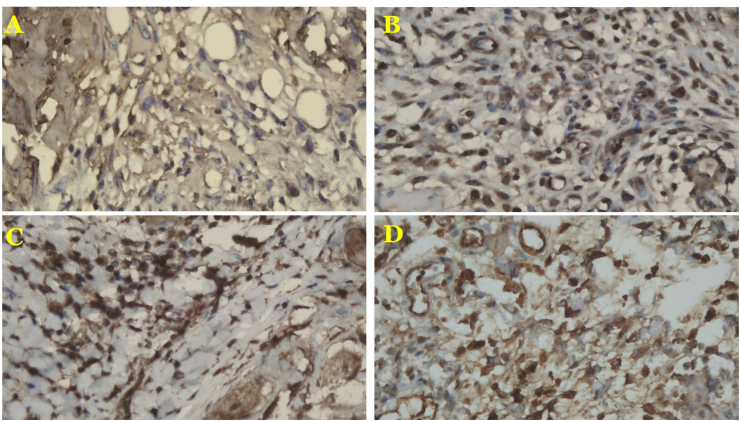
The expressions of *collagen III* on: POD 4 in the monofilament non-absorbable (A) and multifilament absorbable group (B); and POD 7 in the monofilament non-absorbable (C) and multifilament absorbable group (D). The expressions of *collagen III* were significantly higher in the multifilament absorbable group than in the monofilament non-absorbable group on POD 4 (*p* = 0.0003) and 7 (*p* = 0.0004).

All samples were incised in the Anatomy Department of Universitas Gadjah Mada. These experimental procedures were performed with the prior approval of the Medical and Health Research Ethics Committee of the Faculty of Medicine, Public Health and Nursing, Universitas Gadjah Mada/Dr. Sardjito Hospital (KE/FK/0151/EC/2018). All skin samples were incised in full thickness with a rectangular shape (6 × 3 cm). The dermal flaps were then raised, and a 5-cm midline incision in the fascia was made. All samples were divided into 4 groups using a simple random sampling method to determine which group they belong. Group 1 consisted of rats sutured with monofilament non-absorbable (*polyvinylidene fluoride*) large stitch intervals and continuous techniques and were euthanized on postoperative day (POD) 4. Group 2 consisted of rats sutured with multifilament absorbable (*polyglycolide*) large stitch intervals and continuous techniques and were euthanized on POD 4. Group 3 consisted of rats sutured with monofilament non-absorbable (*polyvinylidene fluoride*) large stitch intervals and continuous techniques and were euthanized on POD 7. Group 4 consisted of rats that sutured with multifilament absorbable (*polyglycolide*) large stitch intervals and continuous techniques and were euthanized on POD 7. After euthanasia, samples of fascia that contained the stitches were taken (1 × 0.5 cm) and stained with collagen I and III using immunohistochemistry methods (Fig. 1, Fig. 2). Collagen I and III were then measured using an Adobe Reader counter. We did not sample any different areas to make a comparison.

### 2.3 Statistical analysis

Independent t-tests were used to evaluate the association between monofilament non-absorbable sutures and multifilament absorbable sutures with the collagen I/III ratios. For statistical analyses, a *p* value of less than 0.05 was considered to be significant and 95% confidence interval (CI) was used in this study.

## 3 Results

The expression of *collagen I* in rats sutured with monofilament non-absorbable and multifilament absorbable POD 4 was 41.76 ± 9.09 and 38.43 ± 10.13, respectively (*p* = 0.45), while the expression of *collagen I* in rats sutured with monofilament non-absorbable and multifilament absorbable at POD 7 was 59.23 ± 6.32 and 59.90 ± 6.16, respectively (*p* = 0.81) (Table 1).

**Table 1:** Comparison of *collagen I* and *III* expression between monofilament non-absorbable and multifilament absorbable on PODs 4 and 7.

	*Collagen I* expressions		p	*Collagen III* expressions		p
	Monofilament non-absorbable (mean ± SD)	Multifilament absorbable (mean ± SD)		Monofilament non-absorbable (mean ± SD)	Multifilament absorbable (mean ± SD)	
Day 4	41.76 ± 9.09	38.43 ± 10.13	0.45	48.26 ± 8.82	72.53 ± 14.88	0.0003^a^
Day 7	59.23 ± 6.32	59.90 ± 6.16	0.81	36.63 ± 11.08	61.50 ± 14.49	0.0004^a^

a , *p* < 0.05 is considered significant; SD, standard deviation; POD, postoperative day.

Interestingly, the expression of *collagen III* was significantly different between rats sutured with monofilament non-absorbable and multifilament absorbable either at POD 4 (48.26 ± 8.82 *vs.* 72.53 ± 14.88; *p* = 0.0003) and POD 7 (36.63 ± 11.08 *vs.* 61.50 ± 14.49, *p* = 0.0004) (Table 1).

Moreover, the *collagen I/III* ratio was also significantly different between the two groups at POD 4 (0.88 ± 0.23 *vs.* 0.53 ± 0.08; *p* = 0.0003) and 7 (1.77 ± 0.65 *vs.* 1.03 ± 0.28; *p* = 0.004) (Table 2).

**Table 2:** Comparison of the *collagen I/III* ratio between monofilament non-absorbable and multifilament absorbable on PODs 4 and 7.

	*Collagen I/III* ratio		p
	Monofilament non-absorbable (mean ± SD)	Multifilament absorbable (mean ± SD)	
Day 4	0.88 ± 0.23	0.53 ± 0.08	0.0003^a^
Day 7	1.77 ± 0.65	1.03 ± 0.28	0.004^a^

a , *p* < 0.05 is considered significant; SD, standard deviation; POD, postoperative day.

## 4 Discussion

We are able to show that the *collagen I/III* ratio was significantly higher in the monofilament non-absorbable group than in the multifilament absorbable group. A similar study conducted in 2007 found that non-absorbable monofilament generates greater strength than multifilament absorbable, but this research did not specifically examine collagen existence and amount [8]. A study in National Chen Kung Hospital supported this study and produced similar results. Erasmus University Medical Centre in Rotterdam also conducted a similar study and reached the conclusion that incisional hernia incidence would be greatly reduced by using slowly absorbable materials [9]. Therefore, we aimed to investigate this phenomenon from a different point of view, which is based on the expression of collagen I/III ratios. However, in a study conducted in 1993, incisional hernia incidence was higher in patients sutured with monofilament sutures with continuous techniques (8%) than in patients sutured with multifilament sutures with interrupted techniques (6%) [5]. This contradictive result might be caused by method bias in the latter study mentioned.

Several studies also compared absorbable and non-absorbable sutures. Research in 2015 declared the superiority of absorbable suture materials because they had a lower incidence of suture reactions than non-absorbable suture materials. This research was conducted in Achilles tendon repair cases [10]. While our result is similar to several studies that found that non-absorbable sutures provide greater strength and lower incidence of suture reaction, research conducted in at the University of Western Ontario stated otherwise. Non-absorbable sutures with continuous techniques decreased the incidence of incisional hernia compared with absorbable sutures [4]. In an adult human study with clean wounds of the face or neck, there was no difference in the outcomes between permanent or absorbable suture material [11]. The same result was provided by a study in children who suffered traumatic lacerations. Absorbable material outcomes were as good as those of non-absorbable materials. There was no difference in wound dehiscence found or infection between these two materials [12]. A meta-analysis conducted in 2007 also stated that there is no difference in effectiveness between absorbable and non-absorbable suture materials [13]. If there is no difference in outcome, absorbable materials are preferably used because they can save the surgeon time and lessen patient anxiety and discomfort.

Collagen type III along with collagen type I are fibril forming collagens secreted by fibroblasts. Unlike collagen type I, collagen type III has less firm structure and strength [14]. Collagen type III values surge in the early process of wound healing, especially in the hemostasis phase and early inflammatory phase. Meanwhile, collagen type I, which has a stronger structure and strength, increases slowly but steadily over the same period of time, substituting collagen type III [6]. Therefore, disruption in collagen metabolism would affect fascial wound healing, and furthermore result in occurrences of incisional hernia. Decreases in the collagen I/III ratio are usually used as a marker for diagnosing incisional hernia. Higher collagen I/III ratios accelerate the wound healing process [15]. Delays in the decline of collagen III and slow increases of collagen I might represent delayed wound healing.

The fascia can more efficiently extract and activate cellular elements that are important for the proliferative phase of the wound healing process, such as fibroblast cells and endothelial cells. The observation of fibroblast cellularity shows increases during the 7th day and the 14th day of the fascia incision. Increased collagen staining of the fascia incision signifies the acceleration of the fascia healing process, involving not only earlier fibroblast activation but also earlier induction of collagen synthesis. In addition, at 5–6 h after the wound, there will be an inflammatory phase. In this phase, inflammatory cells secrete cytokines and growth factors to trigger fibroblasts and vascular endothelial cells, which are then used during the proliferative phase. In the final half of the proliferative phase, approximately 2–3 days after the initial wounding, the granulation tissue becomes shrunken [16].

The effect of the technique and material of abdominal fascia incision wound closure by using experimental animals has also been examined. Laboratory mice that are often developed and used in research are albino Wistar rats (*Rattus norvegicus*). Mice are often used as a research model because the characteristics of physiology, biology, and genetics are very similar to humans.

Incisional hernia currently remains a great problem for surgeons. Most of the previous studies related to fascial healing were conducted euthanasia at the end point of the inflammation stage and proliferative stage, or in the other words, at day 7th and later [17]. On the other hand, it is very rare for a surgeon to treat inpatients for more than 7 days. In other words, a definitive marker for predicting incisional hernia has not yet been established. In this study, we euthanized rats on POD 4 and POD 7 and found that this study could be applied more easily in clinical settings.

It should be noted that the amount of type I collagen remains similar between the two suture materials, but the amount of type III collagen increased significantly in the “absorbable” group. The latter contributes to the ratio difference that helped in drawing the conclusions in our study. These facts should be considered during the interpretations of our study.

Our study has some limitations. Several factors might cause the development of incisional hernia, including wound tension, increased abdominal pressure, malnutrition, and suture material, among others. Besides these limitations, the animal model in our study is not a model of incisional hernia. Therefore, there exists a large gap between our findings and their potential application in clinical practice.

## 5 Conclusions

Monofilament non-absorbable sutures show a significantly higher *collagen I/III* ratio than multifilament absorbable for abdominal fascia closure in rats. Our findings imply that the usage of monofilament non-absorbable sutures might have a beneficial effect on decreasing the incisional hernia occurrence.

## Provenance and peer review

Not commissioned, externally peer-reviewed.
